# Supplementary material for: State-dependent modulation of spiny projection neurons controls levodopa-induced dyskinesia in a mouse model of Parkinson’s disease
Source: bioRxiv. 2025 Jan 7:2025.01.02.631090. Preprint. [Version 2] doi: 10.1101/2025.01.02.631090 (PMC11741361; doi:10.1101/2025.01.02.631090)

bioRxiv preprint doi: https://doi.org/10.1101/2025.01.02.631090; this version posted January 7, 2025. The copyright holder for this preprint (which was not certified by peer review) is the author/funder, who has granted bioRxiv a license to display the preprint in perpetuity. It is made available under aCC-BY 4.0 International license.

Supplemental Figure Legends:

Supplemental Figure 1. The frequency of Sr^{2+}-oEPSC was unaltered between off- and on-states in both dSPNs and iSPNs

(A) Box plot summary of Sr^{2+}-oEPSC frequency in off- and on-state dSPNs (off-state, n = 10 cells from 4 mice; on-state, n = 8 cells from 4 mice). n.s., not statistically significant, Mann-Whitney test.

(B) Box plot summary of Sr^{2+}-oEPSC frequency in off-state and on-state iSPNs (off-state, n = 7 cells from 4 mice; on-state, n = 8 cells from 4 mice). n.s., not statistically significant, Mann-Whitney test.

Supplemental Figure 2. ACh release evoked by burst stimulation was elevated in 6-OHDA lesioned and LID off-state mice and suppressed by D2R agonists

Box plot summary of $GRAB_{ACh3.0}$ signals evoked by burst stimulation in unlesioned, 6-OHDA lesioned and LID off-state mice. Same as with single stimulation, ACh release evoked by burst stimulation (20 pulses at 20 Hz) was significantly elevated in 6-OHDA lesioned and LID off-state mice. Bath application of DA (50 nM) or quinpirole (10 μM) strongly suppressed ACh release (unlesioned, n = 13 ROIs from 3 mice; 6-OHDA, n = 10 ROIs from 3 mice; off-state, n = 12 ROIs from 5 mice). **** $p < 0.0001$, *** $p < 0.001$, ** $p < 0.01$, n.s., not statistically significant, Mann-Whitney test for unpaired data and Wilcoxon for paired data.

Supplemental Figure 3. Genetic perturbation of M1R-CDGI signaling in iSPNs increases the therapeutic effect of levodopa in mice with established LID

(A) Box plot summary of the number of contralateral rotations (in 30 s) recorded 40 min after the first and fifth levodopa administration in wildtype or CDGI KO mice (wildtype n = 8 animals; CDGI KO, n = 9 animals). * $p < 0.05$, n.s. not statistically significant, Mann-Whitney test. The session 5 data were the same as in Fig. 7I.

bioRxiv preprint doi: https://doi.org/10.1101/2025.01.02.631090; this version posted January 7, 2025. The copyright holder for this preprint (which was not certified by peer review) is the author/funder, who has granted bioRxiv a license to display the preprint in perpetuity. It is made available under aCC-BY 4.0 International license.

(B) Box plot summary of the number of contralateral rotations (in 30 s) recorded 40 min after the first or fifth levodopa administration in control or M1R CRISPR mice (control n = 11 animals; M1R CRISPR, n = 10 animals). ** $p < 0.01$, n.s. not statistically significant, Mann-Whitney test.

Supplemental Figure 4. Functional validation of M1R CRISPR

(A) Example traces of somatic voltage recordings in response to a 180-pA current injection from iSPNs expressing M1R CRISPR or gRNA alone (control) before and after application of oxo-M. Scale bars are 40 mV and 100 ms.

(B) Box plot summary of the effect of oxo-M on the number of action potentials (APs) evoked (control, n = 7 cells from 3 mice; M1R CRISPR, n = 6 cells from 3 mice). The increase in somatic excitability by oxo-M was prevented by M1R CRISPR. * $p < 0.05$, Mann-Whitney test.

(C) Box plot summary of the effect of oxo-M on rheobase. * $p < 0.05$, Mann-Whitney test.

Supplemental Figure 5. Density of spines with larger head diameters detected by high-resolution confocal microscopy

Box plot summary of the density of spines with > 0.4 μm head diameter in proximal dendrites of sparsely labeled iSPNs imaged by high-resolution confocal microscopy (control: n = 18 dendrites from 4 mice; 6-OHDA: n = 22 dendrites from 3 mice; off-state: n = 25 dendrites from 4 mice; on-state: n = 22 dendrites from 4 mice). This result was similar to the total spine density detected by 2PLSM (Fig. 4B). *** $p < 0.001$, ** $p < 0.01$, * $p < 0.05$, Mann-Whitney test.

bioRxiv preprint doi: https://doi.org/10.1101/2025.01.02.631090; this version posted January 7, 2025. The copyright holder for this preprint (which was not certified by peer review) is the author/funder, who has granted bioRxiv a license to display the preprint in perpetuity. It is made available under aCC-BY 4.0 International license.

Supplemental Figure 1

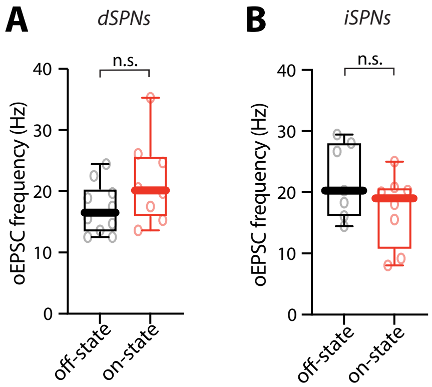

bioRxiv preprint doi: https://doi.org/10.1101/2025.01.02.631090; this version posted January 7, 2025. The copyright holder for this preprint (which was not certified by peer review) is the author/funder, who has granted bioRxiv a license to display the preprint in perpetuity. It is made available under aCC-BY 4.0 International license.

Supplemental Figure 2

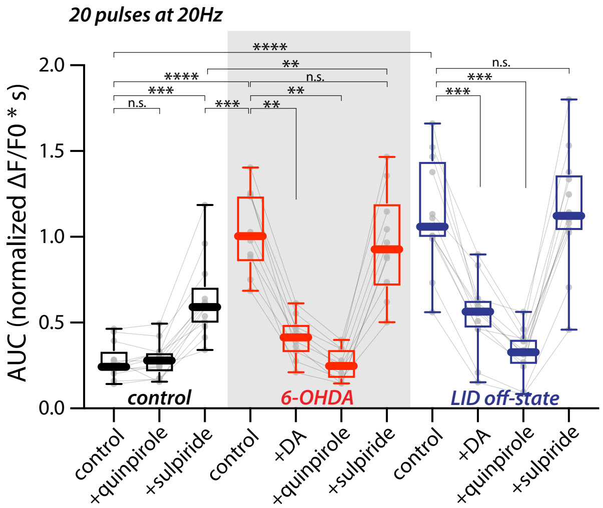

bioRxiv preprint doi: https://doi.org/10.1101/2025.01.02.631090; this version posted January 7, 2025. The copyright holder for this preprint (which was not certified by peer review) is the author/funder, who has granted bioRxiv a license to display the preprint in perpetuity. It is made available under aCC-BY 4.0 International license.

Supplemental Figure 3

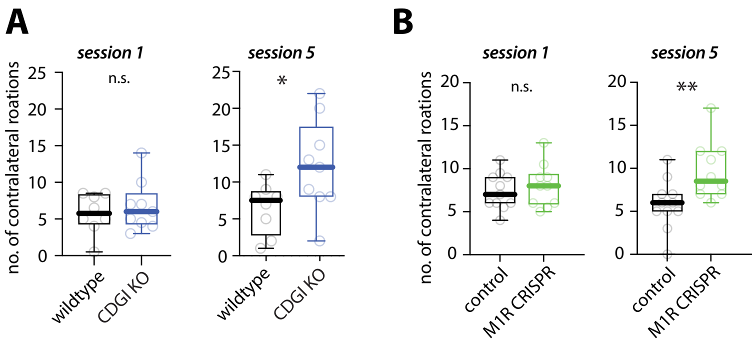

bioRxiv preprint doi: https://doi.org/10.1101/2025.01.02.631090; this version posted January 7, 2025. The copyright holder for this preprint (which was not certified by peer review) is the author/funder, who has granted bioRxiv a license to display the preprint in perpetuity. It is made available under aCC-BY 4.0 International license.

Supplemental Figure 4

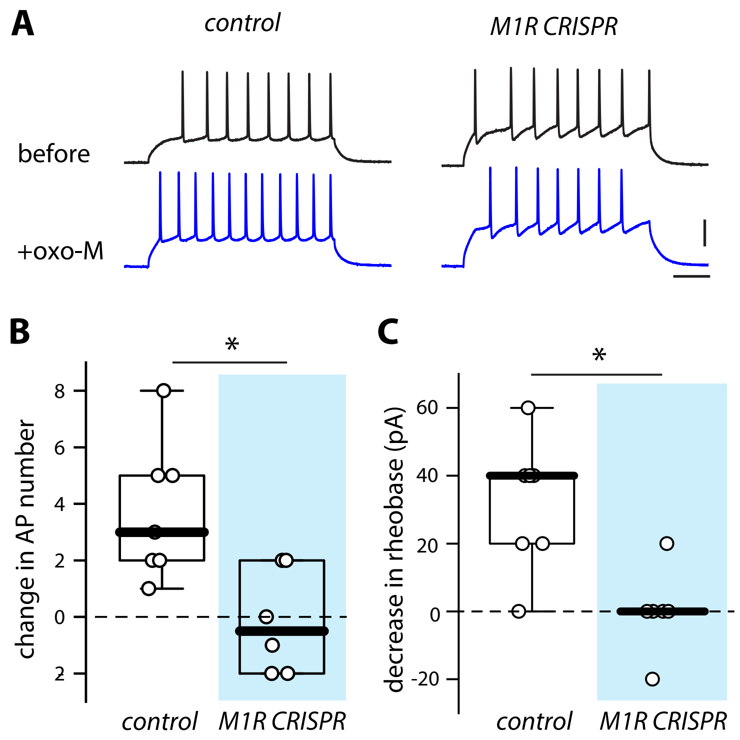

bioRxiv preprint doi: https://doi.org/10.1101/2025.01.02.631090; this version posted January 7, 2025. The copyright holder for this preprint (which was not certified by peer review) is the author/funder, who has granted bioRxiv a license to display the preprint in perpetuity. It is made available under aCC-BY 4.0 International license.

Supplemental Figure 5

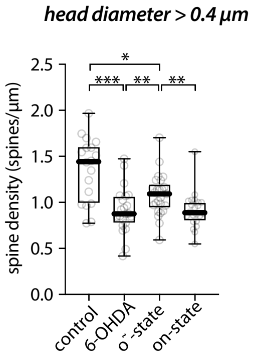

Supplement: Supplement 1 [file NIHPP2025.01.02.631090v2-supplement-1.pdf]
